# Supplementary material for: Conditional chemoconnectomics (cCCTomics) as a strategy for efficient and conditional targeting of chemical transmission
Source: eLife. 2024 Apr 30;12:RP91927. doi: 10.7554/eLife.91927 (PMC11060718; doi:10.7554/eLife.91927)
Supplement: Figure 3—source data 3. [file elife-91927-fig3-data3.zip › Figure 3 source data3 part 2.zip!/Figure 3-Source data 3-part2/1053f2-1-CRISPResso_Report_y3hlioj1.zip!/CRISPResso_on_1053f2_4/2b.Nucleotide_percentage_quilt_around_sgRNA_tggtcctgaccatctacatt.pdf]

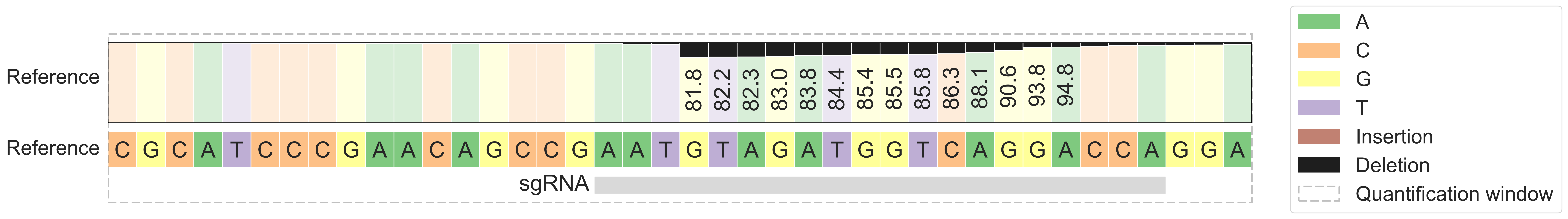

Reference
Reference
C G C A T C C C G A A C A G C C G A A T G T A G A T G G T C A G G A C C A G G A
81.8
82.2
82.3
83.0
83.8
84.4
85.4
85.5
85.8
86.3
88.1
90.6
93.8
94.8
sgRNA
A
C
G
T
Insertion
Deletion
Quantification window
